# Exploring the Factors Influencing the Non‐Prescription Use of Medication in Bangladesh: A Cross‐Sectional Study

**DOI:** 10.1002/hsr2.72755

**Published:** 2026-06-30

**Authors:** Sumaia Rahman, S. M. Mahadi Hasan, Md. Omar Faruk, Md. Aminul Islam Nur, Mst. Hajera Khatun

**Affiliations:** ^1^ Department of Computer Science and Engineering, School of Engineering Varendra University Rajshahi Bangladesh; ^2^ Department of Pharmacy, School of Science and Technology Varendra University Rajshahi Bangladesh

**Keywords:** Bangladesh, non‐prescription medication, prescription‐free, self‐medication

## Abstract

**Background and Aims:**

Use of non‐prescription drugs is a significant health concern for the population in Bangladesh. The ease of access to medicines, the high price of healthcare and lack of information on how to take the drugs properly promote such a practice. The purpose of the study was to single out the demographic, behavioral, and knowledge‐related variables connected with taking medicines without a doctor's prescription.

**Methods:**

A cross‐sectional survey was carried out on 504 participants through online and face‐to‐face data collection. The questionnaire consisted of demographic data, behavior related to medication use, and knowledge concerning medicine regulations. The data was summarized using descriptive statistics. The Chi‐square test was used to test the relationship between variables. The use of multinomial logistic regression (MLR) analysis revealed predictors that are significant. Odds ratios (OR) with 95% confidence intervals (CI) were provided, with *p* < 0.05 being the statistical significance level.

**Results:**

One in five respondents had taken over‐the‐counter medicines owing to the high cost of healthcare. Approximately one out of every four indicated that they could easily obtain medicines without a prescription in pharmacies. The majority of the participants (82.1%) felt that there should be a public education program to ensure that people do not use improper medications. The results of the MLR analysis revealed that self‐diagnosing individuals tended to overdose (OR = 2.806; 95% CI: 1.535–5.129; *p* = 0.001). The use of non‐prescription medications was also linked to poor knowledge regarding proper use of medicines (OR = 7.078; 95% CI: 1.448–34.601; *p* = 0.02).

**Conclusion:**

The use of non‐prescription medication is prominent in Bangladesh and is determined by self‐diagnosis, easy access to drugs, and also ignorance. This practice can be minimized by employing public awareness programs and enhanced regulation.

**Abbreviations**
AMR: antimicrobial resistance
GERD: gastroesophageal reflux disease
IBM: International Business Machines
MLR: multinomial logistic regression
NSAIDs: non‐steroidal anti‐inflammatory drugs
OR: odds ratio
SPSS: Statistical Package for the Social Sciences

## 1 Background

Self‐medication is when a person takes medication without consulting a physician. Non‐prescription means the use of medications normally prescribed but taken without following the doctor's directions. Self‐medication refers to any time a person uses a drug for the purpose of self‐diagnosis or self‐treatment. The benefits of self‐medication depend on whether it is used properly, since using medications improperly can have adverse effects on health [1]. Prescription drugs globally abused are also identified as a worldwide public health issue and are harmful to many populations at large [2]. There is an increasing global concern about the inappropriate use of medicines when there is no physician involvement. The fact that medicines are easy to obtain and lack adequate information describing their use (as well as some people self‐diagnosing) has resulted in the increasing trend of using medicines inappropriately in Bangladesh [3, 4]. Other types of populations in Bangladesh have also reported high rates of non‐prescription or over‐the‐counter medicine, and these studies have shown that self‐medication is common throughout the world [5].

According to previous studies, many retail drug shops in Bangladesh are operated by individuals who are not licensed pharmacists and often lack formal training in pharmacy practice. In many cases, these individuals are shop owners or attendants with limited or no formal education in healthcare or pharmacy, although national regulations require dispensing to be supervised by qualified personnel. This situation is more common in rural and semi‐urban areas, where enforcement of regulations is limited and access to qualified pharmacists is constrained. These people, most of whom are the owners of shops or shop assistants and are selling medicines in most of the community settings without any formal qualification as a professional, and some drug shops are even without proper licenses [3, 6, 7]. Though the national laws mandate prescription‐only medicines to be dispensed by qualified pharmacists, the application of the legislation is usually lax, especially in rural and semi‐urban regions. Such a condition can lead to inappropriate practices of dispensing, failure to counsel patients, giving wrong dosage instructions, and a higher probability of antimicrobial resistance. Community pharmacy practices may also influence antibiotic purchasing behavior and contribute to the inappropriate use of medicines [8]. To focus on the issues raised, the Government of Bangladesh has passed the Drugs and Cosmetics Act 2023, which prohibits the sale of prescription medicines without the proper prescription and has a punishment against the violations. Nevertheless, there are issues of enforcement and monitoring left. In Bangladesh, people are not aware that taking medicines improperly can be dangerous; it's common for them not to know how much medication they should take, any side effects to watch out for, and that they should seek medical advice from a qualified practitioner before using a prescription medication. Previous research has also indicated that the general public relies heavily on purchasing over‐the‐counter medications without a prescription [9]. Various types of medication misuse, amongst others, have resulted in the inappropriate use of antibiotics, which has grown to be one of the biggest problems of public health in Bangladesh. Antibiotics and other medications are often obtained and used without a valid prescription from a registered physician, as they are frequently sold or dispensed without proper authorization, particularly for mild or self‐limiting conditions. These practices are a contributor to antimicrobial resistance (AMR), thus complicating the treatment of the infection and causing the healthcare system to overload [10, 11, 12, 13]. The inappropriate use of antibiotics can also cause failure in treatment, prolonged illness, high costs of healthcare, and adverse drug reactions [14, 15, 16, 17, 18]. Besides regulatory factors, structural problems within the healthcare system also contribute to the misuse of antibiotics and other medications. Access to affordable healthcare services is also limited, which also adds to the use of medicines without a doctor's prescription in Bangladesh. People, especially in rural and semi‐urban areas, face various health risks and various inconveniences due to the lack of trained doctors and inadequate health services. Due to the doctor's consultation fee, people from the middle class and especially the lower class in Bangladesh are not interested in going to the doctor. Then they resort to local pharmacists and buy and consume medicines. Consequently, the community pharmacies in most cases become the initial source of contact for health‐related issues, which can lead to the possibility of self‐medication [6, 7, 19, 20]. The attitudes of the culture toward medicines can also shape self‐medication in Bangladesh. Medicines tend to be viewed as the fast cure to the usual ailment, and thus, this often causes individuals to take antibiotics or analgesics without consulting medical practitioners [21, 22, 23, 24]. However, notwithstanding some of the studies in Bangladesh that have found the prevalence and causes of self‐medication, many of the studies were primarily limited to certain population groups, such as university students or hospital patients. Consequently, there is still a paucity of evidence among the general population. Previous studies have primarily reported prevalence and descriptive findings, while statistically significant predictors identified through multivariate models have been less frequently examined. Thus, the paper focuses on cross‐tabulation and multinomial logistic regression analysis of demographic, behavioral, and knowledge‐based determinants of using non‐prescription medications by the general population of Bangladesh. The findings can be used to educate people about political awareness campaigns and reinforce the policies on pharmacy regulation in Bangladesh by identifying some of the key predictors of the same, including self‐diagnosis, simplified access to medicines, and lack of knowledge on proper medication use.

## 2 Objectives of the Research

This study aims to investigate the significant factors behind why people in Bangladesh use medication without a doctor's prescription.

*Research Questions:* The study is conducted to answer the following questions:

1. Which factors significantly affect people to use medicine without a doctor's prescription?
2. Why do people in Bangladesh use medicines without a doctor's prescription?

## 3 Methods

### 3.1 Study Design

**FIGURE 1:**
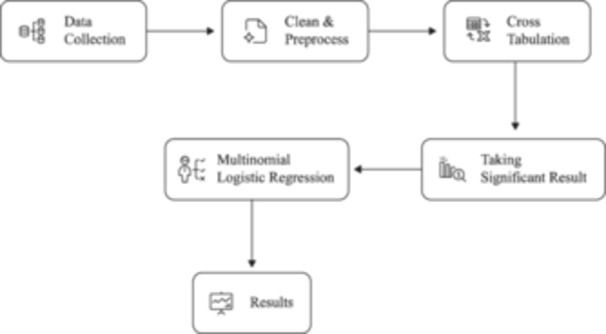
Workflow diagram.

The current study used a cross‐sectional design to establish the reason behind individuals using medicine without a doctor's prescription, which is in line with other normal observational epidemiological designs. The questionnaire contained a number of questions, including demographic data of the participants, medication use, and their behavior, awareness, and knowledge on the issue of medication use, interventions, and the control of the process of taking medicine without a doctor's prescription. The collection of data was done through the use of online and offline methods. The data were gathered in the period between March and June 2025 through online and offline. Google Forms were used as the online survey tool, and the link was sent to the potential participants through social media and direct messaging. Face‐to‐face data collection was conducted in selected urban and rural areas of North Bengal, Bangladesh. These areas were chosen to capture responses from both urban and rural community settings. Data collectors, who were trained, were going to potential participants in the study in public places and community areas to discuss the study objectives. The overall workflow of the study is presented in Figure 1. The way of participation was voluntary. All the participants were made aware of the aims of the study, the confidentiality of their information, and their right to withdraw before giving their answers to the questionnaire. No identifiable personal data was used, and the questionnaire was anonymous. The questionnaire was formulated on the existing literature published on self‐medication and the use of non‐prescription drugs [25, 26, 27]. The initial questionnaire was reviewed by subject‐matter experts in pharmacy and public health to ensure content validity, relevance, and appropriateness of the items. Feedback from the experts was used to refine the wording, structure, and domain coverage of the questions. Prior to the main survey, a pilot study involving 30 participants was conducted to assess clarity, cultural appropriateness, and reliability of the questionnaire, and minor revisions were made accordingly. Pilot responses were made on how to word things and to refine the logical flow of the questions. The responses of the pilots were not analyzed in the end. The non‐probability convenience sample was employed to recruit the participants. The eligibility criteria included individuals 15 years and above from the general population of Bangladesh, who were able to make informed consent about the study by comprehending the questions in the questionnaire. The participants were contacted using online survey links, which were sent via social media and messaging websites, as well as by face‐to‐face recruitment in the selected urban and rural regions of North Bengal. Those who failed to give informed consent or gave partial responses were not included in the final analysis. Only the members of the general population were used as the study sample, and licensed pharmacists were not subjected to the survey directly. Data about pharmacy practice was gathered depending on the perception and experiences of the participants in terms of accessing medicines without a prescription. In the questionnaire survey, “licensed pharmacists” in Bangladesh refers to persons who have been officially authorized by the government or a professional organization to legally practice as pharmacists. Thus, the current research failed to evaluate the difference in behaviors between licensed pharmacists and informal drug sellers. The data on the pharmacy practice is based on the experiences and perceptions of respondents and not by direct observation or interview with the pharmacy staff. When it came to the online survey, the questionnaire link was distributed through social media and chatting applications, and it was on a voluntary basis. The sampled urban and rural areas of North Bengal in the case of the offline survey were approached directly by the trained data collectors. There was no systematic record of the number of participants invited and the number of participants who had refused to participate; hence, a definite response rate could not be ascertained.

### 3.2 Sample

In this study, we used specific parameters such as the confidence interval and the margin of error. The required sample size was determined using Cochran's formula, which is commonly applied in survey‐based studies involving large populations. As it works especially well in situations with big populations, the sample size was calculated using the following formula [28, 29].

$$n=\frac{z^{2}\mathrm{pq}}{d^{2}}=\frac{1.96^{2}\times 0.5\times (1-0.5)}{0.04365^{2}}=504.0616\approx 504$$

Where in this instance *n* = number of samples, *z* = 1.96 (95% interval), *p* = probability of making a choice = 50% or 0.5 (highly dispersed populations), *q* = 1 − *p*, and *d* = percentage of allowed error = 4.365% or 0.04365. This calculation yielded a sample size of 504.

### 3.3 Informed Consent and Ethical Approval

The Varendra University Ethical Review Committee (Reference No: VU/ERC/2025/006) approved the study protocol, all participants were briefed about the aim of the study, the issue of confidentiality of their information and had an option of withdrawing themselves any time during the study without any repercussions; and the informed consent of the participants was signed in written form and presented to the researcher prior to the commencement of the study.

### 3.4 Measures

**FIGURE 2:**
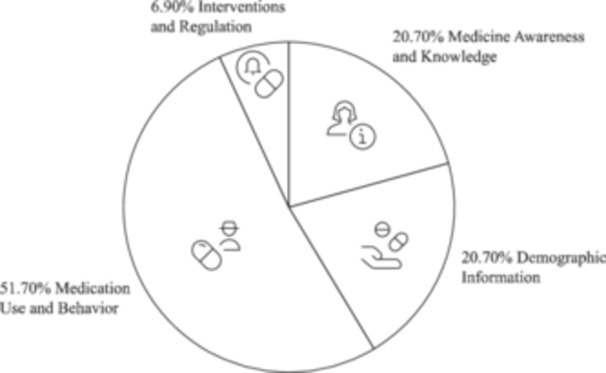
Categories of survey questionnaire.

This survey contained 29 questions, posed to respondents in four categories. These are: demographic information (6 questions/20.70%), questions concerning medical awareness and knowledge (6 questions/20.70%), medication use and behavior (15 questions/51.7%), and interventions and regulation‐related questions (2 questions/6.90%). The majority of the statistics were made in North Bengal, Bangladesh. It was attended by participants in urban and rural regions. Figure 2 shows the structure and categories of the survey questionnaire.

### 3.5 Data Analyses

The results of the survey, both online and offline, were digitized into Microsoft Excel entries for initial organization and validation. A few entries had null values. Since the percentage of missing data was low (less than 5% of the total observations), cases with missing values for the key variables were not excluded from the regression analysis via list‐wise deletion. This method was selected because it will prevent potential bias from mean imputation and maintain the integrity of categorical variables. The information was then entered into IBM SPSS version 25 for further analysis. It was especially appealing for its numerous statistical functions and ease of use, which make it easier to manipulate and interpret complex data. A cross‐sectional bivariate and multinomial logistic regression (MLR) analysis was conducted to establish the significant factors explaining why individuals use medicine without a prescription by a doctor. The bivariate relationship between the dependent and independent variables was tested using cross‐tabulation analysis. The taken *p*‐value was considered statistically significant at 0.05. *p*‐values above 0.05 were considered not to be statistically significant, and mainly odds ratios and 95% confidence intervals were used to interpret the results. The effect sizes were reported as odds ratios (OR) and confidence intervals (CI) of 95%, which indicated the strength and accuracy of the associations [30]. In the multinomial logistic regression (MLR) model, the dependent variable represented the primary reason for using medicine without a doctor's prescription, as reported by the participants. This variable had four categories, namely (a) cost of healthcare, (b) ease of access, (c) lack of awareness, and (d) self‐diagnosis. The reference category for the multinomial logistic regression model was “cost of healthcare,” and all odds ratios were interpreted relative to this category. In this model, an odds ratio (OR) greater than 1 indicates a higher likelihood of reporting a specific reason compared to the reference category, whereas an OR less than 1 indicates a lower likelihood. Therefore, the multinomial model considered the independent variables correlated with various motivation factors for the use of non‐prescription medication (instead of the dichotomous variable use and non‐use). They used the cross tabulation operation between various independent variables like gender, side effects of medicine without a doctor's prescription, pharmacists willing to provide medicine without a prescription and the like. The information is provided in Table 1. Multinomial logistic regression (MLR) analysis was applied because the dependent variable consisted of multiple nominal categories without a natural ordering, allowing comparison of the relative likelihood of each category with respect to a reference group [31, 32]. For the multinomial logistic regression analysis, the dependent variable was defined as the primary reason for using medicine without a doctor's prescription. Multinomial logistic regression (MLR) analysis was performed to estimate odds ratios (OR) with 95% confidence intervals (CI) and to examine factors associated with the different reasons for using medicines without a doctor's prescription, where all estimates were interpreted relative to the reference category. All odds ratios (OR) are indicated using confidence intervals (CI), 95%. Interpretation was preceded by analysis of model assumptions. Variance Inflation Factor (VIF) was used to assess multicollinearity among the independent variables. The VIF of all the variables were below the accepted standard of 5, indicating that multicollinearity was not significant in the regression model. The findings of the multinomial logistic regression analysis are given in Table 2. As a reference group, the cost of Healthcare category was selected. To present all analyses, effect sizes are reported as odds ratios (OR) with 95% confidence intervals (CI) to indicate the level and precision of the estimates. All statistical tests were two‐sided. The level of statistical significance was considered to be two‐tailed, and a *p* below 0.05 was considered statistically significant. *p*‐values were not stressed since emphasis was made on effect sizes and precision estimates. All the analyses were pre‐specified in accordance with the study objectives. No post hoc subgroup tests were done. This study was reported in accordance with the STROBE (Strengthening the Reporting of Observational Studies in Epidemiology) guidelines for cross‐sectional studies. In order to sum up, descriptive data were employed (frequency, percentage, and mean) to classify people involved in this study. The correlations between the nominal variables were tested with the Chi‐square test (cross‐tabulation). Multinomial logistic regression (MLR) was performed to find out the independent variables in the non‐prescription medications used in the research. All statistical analyses were performed using statistical software (IBM SPSS Statistics for Windows, version 25.0 [IBM Corp., Armonk, NY, USA]) and in accordance with the SAMPL guidelines (Statistical Analyses and Methods in the Published Literature).

**TABLE 1:** Cross‐tabulation analysis of factors associated with the primary reasons for using medicine without a doctor's prescription.

Variable name	Variable levels	Use medicine without a doctor's prescription				Column total (%)	*p* value	VIF
		Cost of healthcare (%)	Self‐diagnosis (%)	Ease of access (%)	Lack of awareness (%)			
Gender	Male	107 (21.2)	103 (20.4)	94 (18.7)	29 (5.8)	333 (66.1)	0.70	1.105
	Female	52 (10.3)	48 (9.5)	57 (11.7)	14 (2.8)	171 (33.9)		
Antibiotic without a prescription	No	70 (13.7)	71 (14.1)	74 (14.7)	25 (5)	240 (47.6)	0.41	1.071
	Yes	89 (17.7)	80 (15.9)	77 (15.3)	18 (3.6)	264 (52.4)		
Used listed medicine without a prescription	Occasionally	123 (24.4)	119 (23.6)	123 (24.4)	36 (7.1)	401 (79.6)	0.26	1.089
	Regularly	21 (4.2)	17 (3.4)	15 (3)	3 (0.6)	56 (11.1)		
	Frequently	11 (2.2)	15 (3)	13 (2.6)	4 (0.8)	43 (8.5)		
	Daily	4 (0.8)	0 (0)	0 (0)	0 (0)	4 (0.8)		
Using medicines without prescription is beneficial for health	No	49 (9.7)	47 (9.3)	59 (11.7)	25 (5)	180 (35.7)	0.03	1.062
	Yes	106 (21)	99 (19.6)	89 (17.7)	17 (3.4)	311 (61.7)		
	Others	4 (0.8)	5 (1)	3 (0.6)	1 (0.2)	13 (2.6)		
Side effects using medicines without prescription	Yes	103 (20.4)	115 (22.5)	119 (23.6)	31 (6.2)	368 (73.0)	0.03	1.001
	No	56 (11.1)	36 (7.1)	32 (6.3)	12 (2.4)	136 (27)		
AMR antimicrobial resistance knowledge	Yes	96 (19)	103 (20.4)	93 (18.5)	30 (6.0)	322 (63.9)	0.38	1.163
	No	63 (12.5)	48 (9.5)	58 (11.5)	13 (2.6)	182 (36.1)		
Usually obtain medications without prescription	From pharmacy	114 (22.6)	124 (24.6)	124 (24.6)	124 (6.2)	393 (78)	0.11 (likelihood)	1.150
	From friends/family	30 (6.0)	24 (4.8)	19 (3.8)	8 (1.6)	81 (16.1)		
	From online	13 (2.6)	3 (0.6)	6 (1.2)	3 (0.6)	25 (5.0)		
	Other	2 (0.4)	0 (0.0)	2 (0.4)	1 (0.2)	5 (1.0)		
Licensed pharmacists willing to give medicine without prescription	No	36 (7.1)	24 (4.8)	22 (4.4)	16 (3.2)	98 (19.4)	0.004	1.164
	Yes	123 (24.4)	127 (25.2)	129 (25.6)	27 (5.4)	406 (80.6)		
Full course prescribed medications completion	No	82 (16.3)	75 (14.9)	70 (13.9)	16 (3.2)	243 (48.2)	0.37	3.043
	Yes	77 (15.3)	76 (15.1)	81 (16.1)	27 (5.4)	261 (51.8)		
Reasons for not completing full course of medicine	Completed course	78 (15.5)	82 (16.3)	82 (16.3)	27 (5.4)	269 (53.4)	0.22	2.953
	Feeling better	49 (9.7)	50 (9.9)	44 (8.7)	12 (2.4)	155 (30.8)		
	Forgot	16 (3.2)	13 (2.6)	19 (3.8)	4 (0.8)	52 (10.3)		
	Side effects	15 (3.0)	4 (0.8)	5 (1.0)	0 (0.0)	24 (4.8)		
	Others	1 (0.2)	2 (0.4)	1 (0.2)	0 (0.0)	4 (0.8)		
Medicine disposal place	Dustbin	137 (27.2)	125 (24.8)	110 (21.8)	37 (7.3)	409 (81.2)	0.12	1.082
	Throw outside	17 (3.4)	16 (3.2)	30 (6)	3 (0.6)	66 (13.1)		
	Bury	2 (0.4)	5 (1)	4 (0.8)	2 (0.4)	13 (2.6)		
	Other	3 (0.6)	5 (1)	7 (1.4)	1 (0.2)	16 (3.2)		
Have taken higher‐dose medication than prescribed	No	97 (19.2)	118 (23.4)	125 (24.8)	29 (5.8)	369 (73.2)	0.001	1.321
	Yes	62 (12.3)	33 (6.5)	26 (5.2)	14 (2.8)	135 (26.8)		
How you decide to use medications without prescription	Past experience	60 (11.9)	72 (14.3)	49 (9.7)	11 (2.2)	192 (38.1)	0.28	1.048
	Recommendations from friends/family	43 (8.5)	30 (6.0)	30 (6.0)	16 (3.2)	119 (23.6)		
	Pharmacy advice	34 (6.7)	27 (5.4)	45 (8.9)	7 (1.4)	113 (22.4)		
	Internet research	19 (3.8)	18 (3.6)	26 (5.2)	8 (1.6)	71 (14.1)		
	Other	3 (0.6)	4 (0.8)	1 (0.2)	1 (0.2)	9 (1.8)		
Taking overdosed drug or not	No	91 (18.1)	112 (22.2)	102 (20.2)	23 (4.6)	328 (65.1)	0.005	1.337
	Yes	68 (13.5)	39 (7.7)	49 (9.7)	20 (4.0)	176 (34.9)		
Understand risks associated with misuse medications	Moderately	62 (12.3)	47 (9.3)	57 (11.3)	13 (2.6)	179 (35.5)	0.39	1.143
	Very well	44 (8.7)	59 (11.7)	42 (8.3)	12 (2.4)	157 (31.2)		
	Slightly	40 (7.9)	37 (7.3)	43 (8.5)	13 (2.6)	133 (26.4)		
	Not at all	13 (2.6)	8 (1.6)	9 (1.8)	5 (1.0)	35 (6.9)		
Received education about proper medication use	No	77 (15.3)	49 (9.7)	69 (13.7)	15 (3.0)	210 (41.7)	0.02	1.120
	Yes	82 (16.3)	102 (20.2)	82 (16.3)	28 (5.6)	294 (58.3)		
Misusing medications impacted health	No	48 (9.5)	40 (7.9)	38 (7.5)	12 (2.4)	138 (27.4)	0.78	1.077
	Yes	111 (22.0)	111 (22.0)	113 (22.4)	31 (22.4)	366 (72.6)		
Rate your knowledge about correct use of medicine	Good	55 (10.9)	71 (14.1)	55 (10.9)	14 (2.8)	195 (38.7)	0.002	1.163
	Excellent	45 (8.9)	44 (8.7)	44 (8.7)	12 (2.4)	145 (28.8)		
	Fair	46 (9.1)	34 (6.7)	48 (9.5)	10 (2.0)	138 (27.4)		
	Poor	13 (2.6)	2 (0.4)	4 (0.8)	7 (1.4)	26 (5.2)		
Awareness of prescription medication laws in Bangladesh	No	104 (20.6)	112 (22.2)	104 (20.6)	26 (5.2)	346 (68.7)	0.24	1.208
	Yes	55 (10.9)	39 (7.7)	47 (9.3)	17 (3.4)	158 (31.3)		
Pharmacies regulated to prevent sale medications	No	33 (6.5)	23 (4.6)	20 (4.0)	9 (1.8)	85 (16.9)	0.27	1.119
	Yes	126 (25.0)	128 (25.4)	131 (26.0)	34 (6.7)	419 (83.1)		
Interventions reduce pharmaceutical misuse	Awareness programs	65 (12.9)	80 (15.9)	73 (14.5)	20 (4.0)	238 (47.2)	0.20	1.059
	Better access to healthcare	53 (10.5)	40 (7.9)	41 (8.1)	8 (1.6)	142 (28.2)		
	Stricter regulations	41 (8.1)	31 (6.2)	37 (7.3)	15 (3.0)	124 (24.6)		
Participate awareness programs about proper medication use	No	26 (5.2)	37 (7.3)	18 (3.6)	9 (1.8)	90 (17.9)	0.03	1.098
	Yes	133 (26.4)	114 (22.6)	133 (26.4)	34 (6.7)	414 (82.1)		
Age grouped	15–22	78 (15.5)	77 (15.3)	74 (14.7)	25 (5.0)	254 (50.4)	0.73	1.308
	23–78	81 (16.1)	74 (14.7)	77 (15.3)	18 (3.6)	250 (49.6)		
Yearly income	0–49,999	69 (13.7)	77 (15.3)	80 (15.9)	18 (3.6)	244 (48.4)	0.26	1.350
	50,000–1,500,000	90 (17.9)	74 (14.7)	71 (14.1)	25 (5.0)	260 (51.6)		
Weight	30–60	74 (14.7)	67 (13.3)	71 (14.1)	22 (4.4)	234 (46.4)	0.88	1.235
	61–100	85 (16.9)	84 (16.7)	80 (15.9)	21 (4.2)	270 (53.6)		
Height group (m)	1.2446–1.6256	60 (11.9)	58 (11.5)	53 (10.5)	16 (3.2)	187 (37.1)	0.93	1.235
	1.651–1.7018	50 (9.9)	42 (8.3)	52 (10.3)	12 (2.4)	156 (31.0)		
	1.72–2.667	49 (9.7)	51 (10.1)	46 (9.1)	15 (3.0)	161 (31.9)		
BMI	15–22.5	78 (15.5)	75 (14.9)	78 (15.5)	25 (5.0)	256 (50.8)	0.74	1.163
	22.51–35	81 (16.1)	76 (15.1)	73 (14.5)	18 (3.6)	248 (49.2)		

**TABLE 2:** Multinomial logistic regression analysis of factors associated with the primary reasons for using medicine without a doctor's prescription.

Variables	Use medicine without a doctor's prescription (Ref = cost of healthcare)											
	Self‐diagnosis				Ease of access				Lack of awareness			
	*p* value	OR	95% CI for OR		*p*‐value	OR	95% CI for OR		*p* value	OR	95% CI for OR	
			Lower bound	Upper bound			Lower bound	Upper bound			Lower bound	Upper bound
Using medicines without a prescription is beneficial for health (ref: Others)												
Yes	0.94	0.953	0.214	4.236	0.54	1.657	0.327	8.405	0.45	2.47	0.239	25.517
No	0.87	0.884	0.205	3.805	0.81	1.216	0.245	6.029	0.80	0.739	0.072	7.606
Side effects of using medicines without a prescription (ref: No)												
Yes	0.53	1.198	0.687	2.088	0.18	1.471	0.836	2.588	0.30	1.55	0.680	3.524
Licensed pharmacists willing to give medicine without a prescription (ref: No)												
Yes	0.24	0.685	0.367	1.281	0.10	0.584	0.310	1.101	0.05	2.27	1.006	5.127
Medicine disposal place (ref: Others)												
Dustbin	0.49	0.580	0.126	2.668	0.09	0.289	0.068	1.223	0.61	0.541	0.051	5.711
Throw outside	0.51	0.571	0.108	3.010	0.46	0.559	0.118	2.648	0.42	0.334	0.024	4.721
Burn	0.69	1.585	0.160	15.694	0.60	0.553	0.057	5.331	0.86	1.34	0.056	31.963
Have taken higher dose medication than prescribed (ref: No)												
Yes	0.07	1.716	0.957	3.077	0.001	2.806	1.535	5.129	0.52	1.33	0.558	3.152
Taking overdosed drug or not (ref: No)												
Yes	0.21	1.438	0.815	2.535	0.83	0.941	0.545	1.625	0.53	0.765	0.331	1.771
Received education about proper medication use (ref: No)												
Yes	0.05	0.611	0.371	1.006	0.88	0.962	0.591	1.566	0.12	0.547	0.258	1.163
Rate your knowledge about correct use of medicine (ref: Excellent)												
Poor	0.02	7.078	1.448	34.601	0.13	2.615	0.753	9.087	0.09	0.348	0.101	1.199
Fair	0.04	5.224	1.049	26.023	0.13	2.644	0.747	9.358	0.07	0.306	0.085	1.106
Good	0.05	4.910	0.984	24.493	0.10	2.818	0.807	9.835	0.06	0.290	0.080	1.053
Participate awareness programs about proper medication use (ref: No)												
Yes	0.02	2.053	1.123	3.751	0.53	0.802	0.406	1.585	0.57	1.31	0.522	3.280

## 4 Results

**FIGURE 3:**
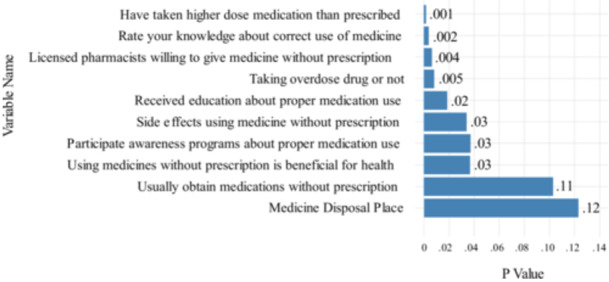
Significant factors identified in the cross‐tabulation analysis of reasons for using medicine without a doctor's prescription.

**FIGURE 4:**
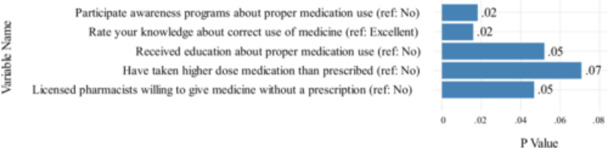
Significant factors identified in the multinomial logistic regression (MLR) analysis of reasons for using medicine without a doctor's prescription.

Table 1 presents the results of the cross‐tabulation analysis examining factors associated with the primary reasons for using medicine without a doctor's prescription. Some of the noteworthy factors identified through the cross‐tabulation analysis were, using medicines without prescription is beneficial to the health, side effects using medicines without prescription, licensed pharmacists willing to give medicine without prescription, have taken higher dose medication than advised, taking overdosed drug or not, received education about proper medication use, rate your knowledge about proper use of medicine and participation in awareness programs on proper medication use. Figure 3 shows the key variables that were found using the cross‐tabulation. Based on cross‐tabulation, the results revealed that 106 (21%) respondents believed that, because of the cost of healthcare, it is healthy to use the medicine without doctor. Out of 504 respondents, 103 (20.4%) participants taking non‐prescribed medicine because of the cost of healthcare experienced the negative side effects of medicine, 115 (22.5%) participants preferred to use their own diagnosis and took non‐prescribed medicine experienced the negative side effects of medicine and 119 (23.6%) participants felt taking non‐prescribed medicine is easy because they took non‐prescribed medicine without the prescription of a registered doctor. Among the respondents who took into consideration the price of healthcare, 123 (24.4%) respondents said it was easier to self‐diagnose, 127 (25.2%) said it was easier to use non‐prescription medication, and 129 (25.6%) said it was easier to use pharmacist‐prescription medication. Of the number of participants 504, 195 (38.7%) participants replied affirming that they knew well how to use medicine, 145 (28.8) participants knew very well, and 138 (27.4%) participants knew fairly how to use medicine. Although they are educated, they take non‐prescribed medicine due to the assumption that it is simple to access medicine without being prescribed, they consider the cost of healthcare, and they like self‐treatment. In the cross‐tabulation study of the total participants, it was discovered that 414 (82.1%) respondents believed that to avert the issue, public education and awareness programs ought to be held concerning the correct usage of medication. Table 2 presents the results of the multinomial logistic regression analysis examining factors associated with the use of medicine without a doctor's prescription. Figure 4 shows the statistically significant factors that were identified in the multinomial logistic regression model. Based on this analysis, respondents found out that medicines were sometimes available in pharmacies without a prescription from a doctor. The regression analysis showed a relationship between perceived pharmacy dispensing practices and a lack of awareness of proper medication taking (OR = 2.27, 95% CI: 1.006–5.127, *p* = 0.05). In addition, no statistically significant association was observed between perceived dispensing practices and contacting the prescribing physician (OR = 0.584; 95% CI: 0.310–1.101; *p* = 0.10). Based on the analysis, no statistically significant association was observed between self‐diagnosis and taking a higher dose of medication (OR = 1.716; 95% CI: 0.957–3.077; *p* = 0.07), although this association was not statistically significant. Participants who reported self‐diagnosis as the primary reason were more likely to report ease of obtaining medicines without visiting a healthcare facility compared to those citing cost of healthcare as the primary reason (OR = 2.806; 95% CI: 1.535–5.129; *p* = 0.001). The association between receiving education about proper medication use and self‐treatment was borderline and should be interpreted with caution (OR = 0.611; 95% CI: 0.371–1.006; *p* = 0.05). This may be because some participants believed that obtaining medicines without a prescription is easy and does not lead to complications. Participants with poor (OR = 7.078; 95% CI: 1.448–34.601; *p* = 0.02) and fair (OR = 5.224; 95% CI: 1.049–26.023; *p* = 0.04) knowledge were more likely to report lack of awareness as the primary reason for using medicines without a doctor's prescription compared to those with excellent knowledge, relative to the reference category. No statistically significant association was observed between lack of awareness and the use of medicines without a doctor's prescription (OR = 0.348; 95% CI: 0.101–1.199; *p* = 0.09). No statistically significant association was observed between fair knowledge and reporting lack of awareness as a reason for prescription‐free use of medicines (OR = 0.306; 95% CI: 0.085–1.106; *p* = 0.07). The respondents in the survey who favored the self‐diagnosis or self‐observation were more inclined to believe that everybody ought to be part of the awareness program regarding the use of medicine properly (OR = 2.053; 95% CI: 1.123–3.751; *p* = 0.02).

## 5 Discussion

This paper aims to explore and establish the noteworthy factors and reasons why the population of Bangladesh self‐treats with medication prescriptions. We have explored and discovered that there are statistically significant factors and non‐significant factors that can be significant in the previous studies. The self‐medication rate of the last year in Chattogram was found to be 41.5%, most of the time due to a perception of mild illness, but based on experience or prior prescription [27]. Sixty‐nine percent of those sampled self‐medicated, with antibiotics being 21% of the total use, based on quick relief and time conditions [25]. Ninety‐three percent of participants are self‐medicated, most commonly with NSAIDs, antacids, antihistamines and antibiotics [4]. And they acquired medicines at local drug stores because of lenient sickness perception and time‐saving. According to another study, the majority of the Bangladeshi population takes medicine without a prescription given by the doctor due to time‐saving, cost‐saving and easy access to the medication; they choose to self‐diagnose or self‐treat the illness [26, 33]. Similar self‐medication practices have also been observed in neighboring countries, indicating that this issue extends beyond Bangladesh and represents a broader regional concern [34]. In our study, no statistically significant association was observed between self‐diagnosis and the use of higher doses of medication (OR = 1.716; 95% CI: 0.957–3.077; *p* = 0.07). The multinomial logistic regression results should be interpreted relative to the reference category (cost of healthcare), which represents the baseline comparison for all reported odds ratios. In contrast, self‐diagnosis was significantly associated with the belief that medicines could be easily obtained without visiting a healthcare facility (OR = 2.806; 95% CI: 1.535–5.129; *p* = 0.001). Previous studies have reported that some pharmacy staff in Bangladesh may lack formal training or appropriate qualifications, and that 96.8% did not ask patients about their medical history prior to dispensing medications [7]. Having 21% of GERD drugs and 38% of antipyretics, self‐medication requests were utilized to obtain them [35]. Some participants of this research stated that in some cases, medicines were available in pharmacies without a doctor's prescription. Regression analysis implied the relationship between the perceived pharmacy dispensing practices and the perceived ease of obtaining medicines without prescription (OR = 0.584; 95% CI: 0.310–1.101), but this relationship was not statistically significant. It was mentioned that 439 students of Chittagong University expressed that they possess very poor and low‐level knowledge of the right procedure of taking medication [36]. Sixty‐eight percent of people participating in the study did not know the adverse side effects of taking medication because they lacked knowledge of taking medications [37]. In another study, 64 out of 209 residential university students in Dhaka reported that they were not aware of the potential adverse effects of painkillers [26]. The participants of our study stated that sometimes it was possible to get medicines in pharmacies without providing a prescription from a doctor. The results of the multinomial logistic regression showed that there was a correlation between perceived pharmacy dispensing practices and the ignorance of the correct use of medication (OR = 2.27; 95% CI: 1.006–5.127; *p* = 0.05). No statistically significant association was observed between perceived access to medicines without prescription verification and the outcome (OR = 0.584; 95% CI: 0.310–1.101; *p* = 0.10). It was also determined through our study that the participants who had preference toward self‐medication/self‐diagnosis possessed very poor knowledge (OR = 7.078; 95% CI: 1.448–34.601; *p* = 0.02) regarding the proper use of medicine. In addition, they were not aware of the way medication should be used (OR = 0.348; 95% CI: 0.101–1.199; *p* = 0.09). Under the Drugs and Cosmetics Act 2023 (Enacted September 18, 2023) [38], only antibiotics and all antimicrobial drugs (antifungals, antivirals, and antiprotozoals) are not allowed to be sold without the prescription of a registered physician. In addition, there is a fine of Tk 20,000 on the pharmacy owners against every violation. In other cases, that are more serious, like selling expired medicine or selling unlicensed medicine, then the proprietors of pharmacies may be sentenced to 10 years' imprisonment and up to 10 lakhs Tk fine. Nevertheless, despite these regulations, some past research studies have found prescription medicines to be dispensed without a valid prescription in community pharmacies in Bangladesh. Participants in the current study reported that medicines could be readily accessed without a prescription. These results can be an indication of structural difficulties, which include regulatory laxity, healthcare prices, obstacles to health care services and gaps in societal consciousness. Qualified and well‐trained pharmacists are very relevant in enhancing the safe and rational use of medicines. Participants in this study reported that medicines can sometimes be obtained from pharmacy staff without a prescription in community settings. A lack of adequately trained pharmacy personnel has been associated with an increased risk of improper dispensing practices, insufficient patient counseling, and misuse of prescription medications, including antibiotics. Enhancing the deployment of qualified pharmacists in community pharmacies, the regulatory monitoring and continuous professional training could assist in the provision of safer dispensing practice and the prevention of the misuse of prescription drugs. There are a number of limitations that can be made to this study when interpreting the findings. To begin with, a cross‐sectional design makes it impossible to determine causal relationships between the determinants and non‐prescription medication use. Second, self‐reported responses were used to collect data, and this could be affected by both recall bias and social desirability bias because the respondents could under‐ or over‐report some behaviors involving the use of medications. Third, online and face‐to‐face methodologies of the survey could have created mode differences in the responses of the respondents. Also, the majority of respondents were recruited in North Bengal, and this could narrow the generalization of the results to other parts of Bangladesh. The future study involving nationally representative samples and longitudinal research would offer a better picture of factors that determine the use of non‐prescription medications. Another limitation is that this study did not directly compare the dispensing behaviors of licensed pharmacists and informal drug sellers. Future research, including direct surveys or observational studies of pharmacy personnel, would provide a clearer understanding of these differences.

## Conclusion

Through this research, we identified the factors that prompt citizens of Bangladesh to take medicine without a doctor's prescription. And our research recommends that the majority of the population in Bangladesh use non‐prescribed medicine due to their self‐medication behavior or self‐diagnosis. Two main factors contribute to the preference for self‐treatment: perceived convenience of self‐diagnosis compared to visiting healthcare facilities, and the desire to reduce both time and healthcare costs. It can save time and reduce healthcare cost. The other one is due to insufficient education or ignorance about how to use medicine. Based on this research, it is possible that non‐prescribed medication use poses significant risks to human health. These results indicate the need to raise public awareness of the correct use of medications and to strengthen regulatory oversight of the dispensing process. Education and access to healthcare services can also help reduce the use of non‐prescription medications. We should also address this issue, and simplify and enhance the healthcare system and make telemedicine more convenient. Ensuring the rational use of medicines is essential for improving public health and reducing inappropriate medication practices [39].

## Author Contributions

**Sumaia Rahman:** writing – original draft, formal analysis, data curation, investigation, methodology. **S. M. Mahadi Hasan:** data curation, investigation, writing – original draft, formal analysis. **Md. Omar Faruk:** writing – review and editing, software, project administration. **Md. Aminul Islam Nur:** writing – review and editing, project administration. **Mst. Hajera Khatun:** conceptualization, formal analysis, supervision, validation, writing – review and editing.

## Funding

The authors have nothing to report.

## Consent

The authors have nothing to report.

## Conflicts of Interest

The authors declare no conflicts of interest.

## Transparency Statement

Mst. Hajera Khatun affirms that this manuscript is an honest, accurate, and transparent account of the study being reported; that no important aspects of the study have been omitted; and that any discrepancies from the study as planned (and, if relevant, registered) have been explained.

## Data Availability

The data that support the findings of this study are available from the corresponding author upon reasonable request.
